# Assisted Snapshot GNSS Position, Navigation, and Timing for LEO Satellites

**DOI:** 10.3390/s26206594

**Published:** 2026-10-17

**Authors:** Seunghwan Kim, Tanner Holmes, Tyler Schmitz, Gabriel Wardall, Dennis Akos

**Affiliations:** 1 Department of Aerospace Engineering, University of Illinois Urbana-Champaign, Urbana, IL 61801, USA; seunghwan.kim@colorado.edu; 2 Smead Aerospace Engineering Sciences, University of Colorado Boulder, Boulder, CO 80309, USA; tanner.holmes03@gmail.com (T.H.); tyler.schmitz@colorado.edu (T.S.); gabriel.wardall@colorado.edu (G.W.); 3 GPS Laboratory, Department of Aeronautics and Astronautics, Stanford University, Stanford, CA 94305, USA

**Keywords:** PNT, SSA, CubeSat, GNSS, aGNSS, LEO, Orbits, SDR

## Abstract

This paper presents a novel conceptual design for a low Size, Weight, Power, and Cost (SWaP-C) snapshot Global Navigation Satellite System (GNSS) positioning system for Low Earth Orbit (LEO) CubeSats to enhance their Space Situational Awareness (SSA). Assisted GNSS (aGNSS) strategies allow for small snapshot collections of GNSS signals to be used for full position, velocity, and timing solutions at low-power usage. Key limitations of commercial GNSS receivers for CubeSats and the benefits of aGNSS strategies are discussed. Conceptual designs for the receiver architecture for power-efficient implementation are presented. Computation times and power usage are tested with common microprocessors for major functions and algorithms utilized in the full implementation. Methodologies for initial orbit determination and predictions are discussed to enable computation of aGNSS solutions in a dynamic LEO environment, with examination of the importance of an accurate drag parameter and fit span considerations. Strategies that allow efficient updates to GNSS ephemerides by utilizing a Commercial Off-The-Shelf (COTS) GNSS receiver are explored. Tests of propagation accuracy and the consistency of aGNSS availability are performed with real GNSS data acquired from a LEO satellite in orbit. Major functionalities are presented for further exploration of a snapshot GNSS system customizable to the needs of a variety of LEO satellites, with a focus on CubeSat class missions.

## 1. Introduction

With the growing number of objects in Low Earth Orbit (LEO), there is an increasing need for accurate orbit determination and prediction for collision avoidance. Improving Space Situational Awareness (SSA), which involves determining the operational environment of space objects, is needed to protect satellites and the overall space environment. Much has been written about the dangers of collisions in space, including the potential loss of access to the space environment due to the Kessler Syndrome [1]. Technological improvements have allowed easier access to space, resulting in small satellites, including CubeSats, being used by research and commercial institutions around the world. These systems are custom-built and typically have low Size, Weight, Power and Cost (SWaP-C).

While this has greatly improved accessibility to space, it has also crowded the space environment. With more objects in orbit, it is increasingly important for satellite operators to incorporate collision avoidance techniques to protect themselves and the system at large. In general, the motion of objects in orbit is predictable due to accurate dynamical models. Other vehicles like aircraft or cars change trajectory often, requiring a high update rate of position solutions to accurately describe their overall motion. Satellite trajectories are dominated by the effects of gravity, with perturbations to this nominal trajectory from maneuvers, drag, solar radiation, and other sources. This means that consistent position solutions are not necessarily needed for most satellites, unless the mission itself requires a high-fidelity trajectory estimate. The effects of drag in LEO are major perturbations, except for maneuvers, and have significant effects on the long-term motion of satellites. Even for a CubeSat whose mission does not require consistent high-accuracy position solutions, the higher fidelity in trajectory available can have major implications for its collision risk in the modern crowded space environment.

The foundation to predict the probability of collision for a satellite is the estimation of its trajectory and the trajectories of all nearby objects. Satellite operators can use this information to determine whether the collision probability is high enough to warrant maneuvering. With higher positional uncertainties, the probability of collision may be inflated, resulting in more avoidance maneuvers. The work of [2] examines this relationship and shows that as the combined positional accuracy decreases, the number of maneuvers required increases in a squared relationship. As an example, they estimate that a combined positional accuracy of 7 km requires 5000 times more avoidance maneuvers than an accuracy of 100 m. For a satellite operator, the accuracies of the surrounding objects cannot be controlled, but minimizing the uncertainty of a satellite’s position can have major effects on its maneuvering requirements.

Thus, a low-cost, power-efficient, and accurate positioning system that is easily implemented into a variety of satellite architectures is needed. The previous literature proposed leveraging snapshot GNSS positioning for space situational awareness [3]. However, the altitude- and velocity-dependent operational limits implemented in many conventional terrestrial COTS GNSS receivers can restrict the availability of onboard GNSS position solutions under LEO conditions, particularly for small satellites and CubeSats [4]. More importantly, to the authors’ knowledge, no commercially available space-qualified GNSS receiver currently provides the short-duration raw-signal snapshot architecture considered in this work, making such receivers unsuitable for the proposed distributed, low-cost snapshot-receiver architecture for SSA. Currently, Two-Line Elements (TLEs), which are regularly updated for publicly cataloged space objects based on tracking observations, provide a widely accessible source of orbital information for object tracking [5]. These TLEs allow for rough orbit determination and prediction through the associated Simplified General Perturbations-4 (SGP4) propagator. However, they have high errors of around 1 km at epoch and grow at 1.5 km per day, which means that TLE-based predictions quickly become insufficient for enabling collision avoidance [6]. In addition, these TLEs require radio transmission to be used on board a satellite, which may be a key limitation to smaller satellites due to power constraints.

Comparatively, the prior study in [3] estimated that its proposed spaceborne snapshot GNSS receiver architecture could achieve a 3D RMS positioning accuracy of approximately 19 m. Previous terrestrial snapshot GNSS studies have demonstrated positioning errors on the order of tens of meters; for example, Wang et al. [7] reported RMSEs of approximately 6 m in both the north and east directions and 13.6 m in the up direction, while Beuchert and Rogers [8] reported a median positioning error of 11 m. Snapshot receivers spend most of their time powered off, powering on at defined intervals to collect short snapshots of GNSS signals. Compared to traditional space-grade GNSS receivers, snapshot receivers provide accurate position solutions at very low power draw [4]. This makes them ideal candidates for CubeSats, enabling an accuracy improvement of two orders of magnitude compared to traditional TLE-based predictions without a major impact on the CubeSat’s SWaP-C [3].

Snapshots of GNSS signals are too short to determine the signal transmission timestamps or the ephemerides of the GNSS satellites, requiring this information to be provided from other sources. However, they do allow for the determination of raw measurements such as the code phase, doppler frequency, and carrier phase, which can be used in conjunction with position and timing estimates along with GNSS ephemerides to provide assisted GNSS (aGNSS) solutions. Much of the background theory for using snapshot positioning is discussed in [3], with this paper furthering this work by focusing on the conceptual design of the receiver and testing key features to show its feasibility on a small satellite. Snapshot receiver technology itself is not novel; an example of one such receiver is called SnapperGPS, which has a mass of ~3 g and a power consumption of 12.6 mAh per year [8]. In addition, a snapshot receiver has previously flown in space as part of the “Falcon Gold” experiment by the US Air Force [9]. In 1998, a microGPS receiver operated in snapshot mode onboard the SNOE micro-satellite [10]. These systems provide examples of the flight heritage of snapshot receivers in orbit, but each is custom built specifically for their applications.

This paper builds upon this concept and presents a conceptual autonomous snapshot GNSS receiver design comprising a GNSS snapshot front end and an onboard processor, designed for integration into a variety of satellite architectures. This design allows for easy access to accurate position solutions to improve collision avoidance for satellites of all types. Key considerations to determine position and time estimates sufficient for aGNSS solutions are discussed. Design choices of data processing and handling are discussed. Results of a low-fidelity orbit propagation scheme to be used as the initial position estimate for further aGNSS processing are presented, showing that aGNSS solutions can be achieved with a 2-week propagation without updating of the nominal trajectory. As a result, consistent position solutions can be delivered with this system implementation at customizable output rates with much higher accuracies than traditional TLE methods.

## 2. aGNSS Background and Receiver Architecture

### 2.1. Ground-Based GNSS vs. Space-Based aGNSS

To explain the motivation for a GNSS snapshot receiver designed for CubeSats, discussions on the shortcomings of traditional GNSS for receivers in space and the benefits of aGNSS are needed. Global Positioning System (GPS) satellites lie in Medium Earth Orbit (MEO), meaning that satellites in lower orbital profiles like LEO are in view of their signals. There are a few key differences between a receiver in orbit and one on the ground, particularly from the high speeds seen in orbit. With higher speeds, a larger doppler effect will be experienced by a receiver, widening the possible received frequency ranges of GPS signals. Many traditional receivers only search for the maximum doppler span from the dynamics on the ground, meaning that in orbit, some visible satellites will be missed due to too narrow of a search span. In addition, fewer satellites may be visible depending on the antenna used, and the specific satellites in view change much quicker due to the orbital motion. Through use of a GNSS simulator and overlaid LEO orbits, it was found that the average time a GNSS satellite will be visible to a LEO satellite is 15 min. On the ground, a single GNSS satellite may be visible for 6 h depending on its elevation angle. With these factors, a traditional COTS receiver may not consistently be able to receive signals from four GNSS satellites required for a traditional solution and is not set up to handle the dynamics in an orbital environment.

Beyond these technical challenges, as mentioned in Section 1, the use of conventional COTS GNSS receivers in LEO can also be limited by altitude- and velocity-dependent operational constraints, while space-capable alternatives may be relatively costly for low-cost missions. This leads to the demand for a GNSS receiver designed for use in LEO, compatible with CubeSats or other small satellite systems generally limited by their SWaP-C. The most important constraint for designing a low SWaP-C GNSS receiver is the power it requires to compute position solutions. To compute GNSS position solutions without the use of a commercial receiver, two main strategies are possible. The first is to create a custom receiver from scratch, which would require tremendous research and development and is not practical. The second and preferred method is through use of a Software-Defined Radio (SDR) with a traditional processor. The SDR implementation adopted in this paper is based on the work of the Radio Frequency and Satellite Navigation Laboratory (RF and SatNav Lab) at the University of Colorado Boulder, which has created and compiled an open-source GNSS SDR [11]. This code can be run on a variety of processors, transforming In-phase and Quadrature (IQ) data into full GNSS solutions. In addition, the SDR can be easily configured with wider doppler search spans, adjustable delay lock loop and phase lock loop bandwidths, coherent and non-coherent integration times, and a variety of other settings to handle the dynamic LEO environment. In testing with a LEO orbit in a GNSS simulator, this SDR has no issue acquiring, decoding, and computing solutions from GNSS signals. Through use of this SDR, a traditional GNSS solution can be obtained in the LEO environment.

However, repeated full collections and traditional GNSS computations with an SDR are not desired for use on a small satellite due to the high power and computational time required. This leads to the benefits of aGNSS, which can greatly decrease the power and computation time needed to generate position and velocity solutions. aGNSS leverages data from other sources to simplify the calculation of GNSS solutions. The theory is covered in detail in [12], but simplified features are presented here for reference. A main requirement for a GNSS solution is the ephemerides of the GNSS satellites. In a traditional solution, these parameters are transferred by the GNSS satellites as part of their navigation data. Decoding satellite ephemerides directly leads to a large quantity of data needing to be processed by the SDR. However, if these parameters were provided by another source, this full collection could be avoided entirely. Pulling these ephemerides from another source is considered an aGNSS practice. This practice, and aGNSS in general, is utilized on a wide range of applications throughout the world, most notably in smartphones. Many smartphones pull long-term satellite ephemerides over the internet, avoiding the need to directly decode these parameters from the GNSS signals. This enables much more power-efficient and faster GNSS solutions. The methods to pull these ephemerides for our system implementation are discussed in Section 3.3, including a method that requires no transmission for an autonomous system, but acquiring these ephemerides are a key element in aGNSS solutions. In addition to these ephemerides, a rough estimate of the position and current time are needed to produce an accurate aGNSS solution. According to [12], an estimate of the receiver’s position within 100 km of accuracy and a time estimate within 1 min of truth are needed for aGNSS solutions. This requirement is more complicated than accessing ephemerides for a small satellite in LEO, requiring methods of orbit determination and prediction with significant drag perturbations. However, with access to this information, only a small “snapshot” of raw IF data must be used with a SDR to compute accurate position and velocity solutions via aGNSS at a small fraction of the computation time. Another benefit of this aGNSS approach is the customizability of the output rates and duty cycles of the system. With an orbital prediction methodology, aGNSS solutions are enabled for long periods of time after an initial full GNSS solution. This means that position solutions can be computed at any variety of different rates up to 1 Hz or with hours in-between. With slower update rates, several components could be inactive for large periods of time. These choices represent a trade space between the consistency of measurements for an increased usage of power that could be adapted to the needs of a variety of satellites.

One aspect to clarify is the initialization of the system. To initialize the system requires a full collection of GNSS signals to compute position and velocity solutions using traditional methods. With these solutions, orbit determination and prediction methods are used to predict the future location of the satellite, enabling aGNSS solutions for an extended period. This initial process uses the most amount of power but then enables the power-efficient computations of aGNSS solutions.

### 2.2. Receiver Architecture

The system presented in this paper is designed to be adaptable to fit the needs and capabilities of a variety of satellite architectures. First, it is assumed that the small satellite system already includes an antenna capable of receiving GNSS signals, but this could easily be included in the snapshot receiver architecture itself. These GNSS signals are then split and transferred to two separate components. The first component is RF front-end integrated circuit chip, which down-converts the received GNSS signals to a nominal intermediate frequency (IF) and digitizes them into IQ samples. For our design, we selected the MAX2769 from Analog Devices (Wilmington, MA, USA) as our radio component based on its configurability and low power usage. This data is passed to a processor, and the SDR code is run to compute GNSS or aGNSS solutions. The raw signal from the satellite antenna is also passed to a commercial GNSS receiver, which allows power-efficient computation of GNSS ephemerides and an accurate time solution. While some conventional COTS receivers may not provide valid position solutions when their altitude- or velocity-dependent operating limits are exceeded, many receivers still output some information such as GNSS ephemerides and timing information. For our implementation, we have selected an Ublox MAX-M10 receiver (Ublox, Thalwil, Switzerland) due to its extremely low power usage and its ability to provide this information in a LEO environment through simulator testing. In addition, this component showed in testing that the code phase information from each satellite can be output in a LEO environment to be used in the aGNSS solution.

**Figure 1:**
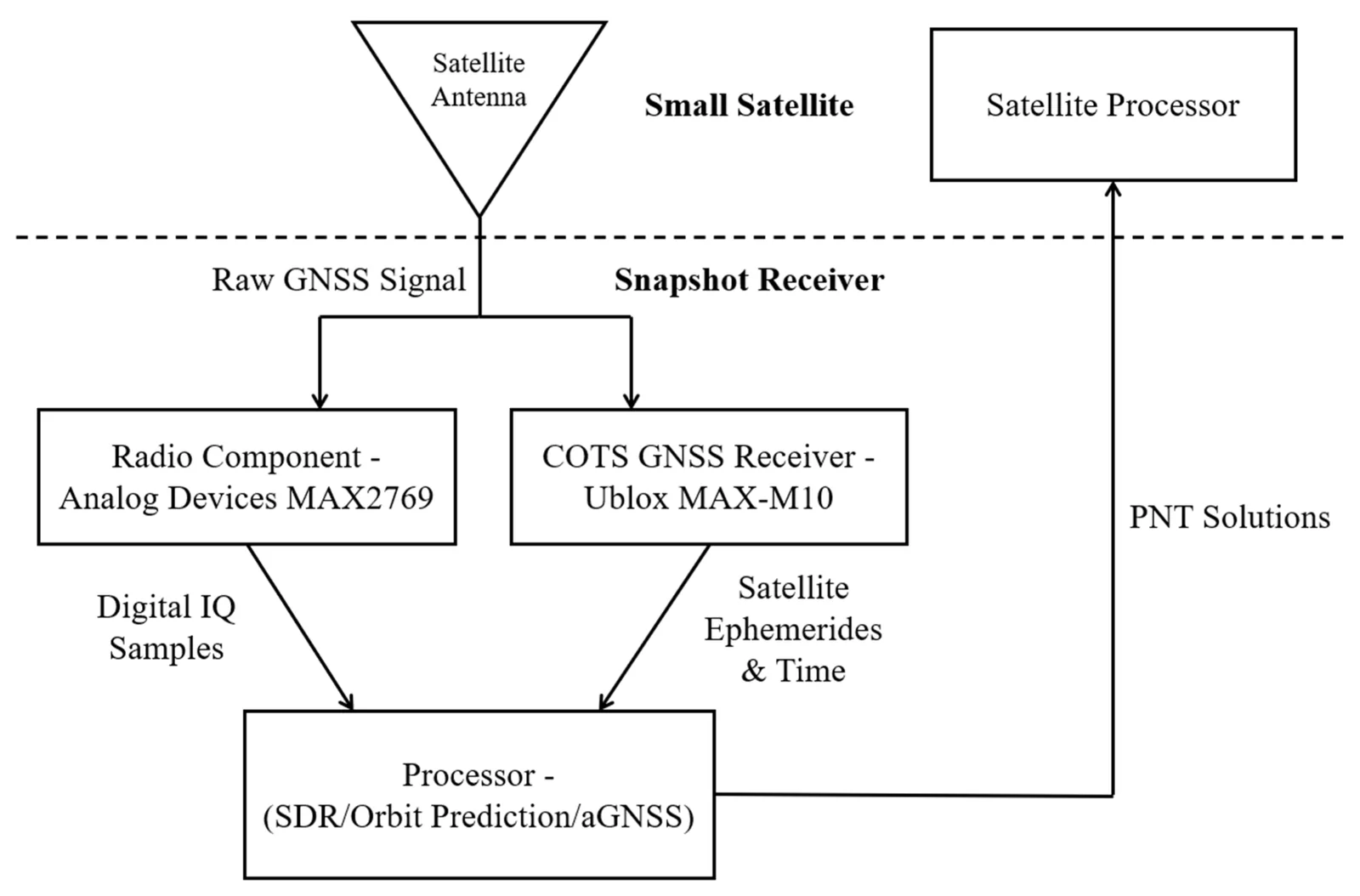
Block diagram of the snapshot receiver architecture featuring an independent processor. aGNSS solutions are computed by the separate processor and passed along to the main processor on the satellite system.

**Figure 2:**
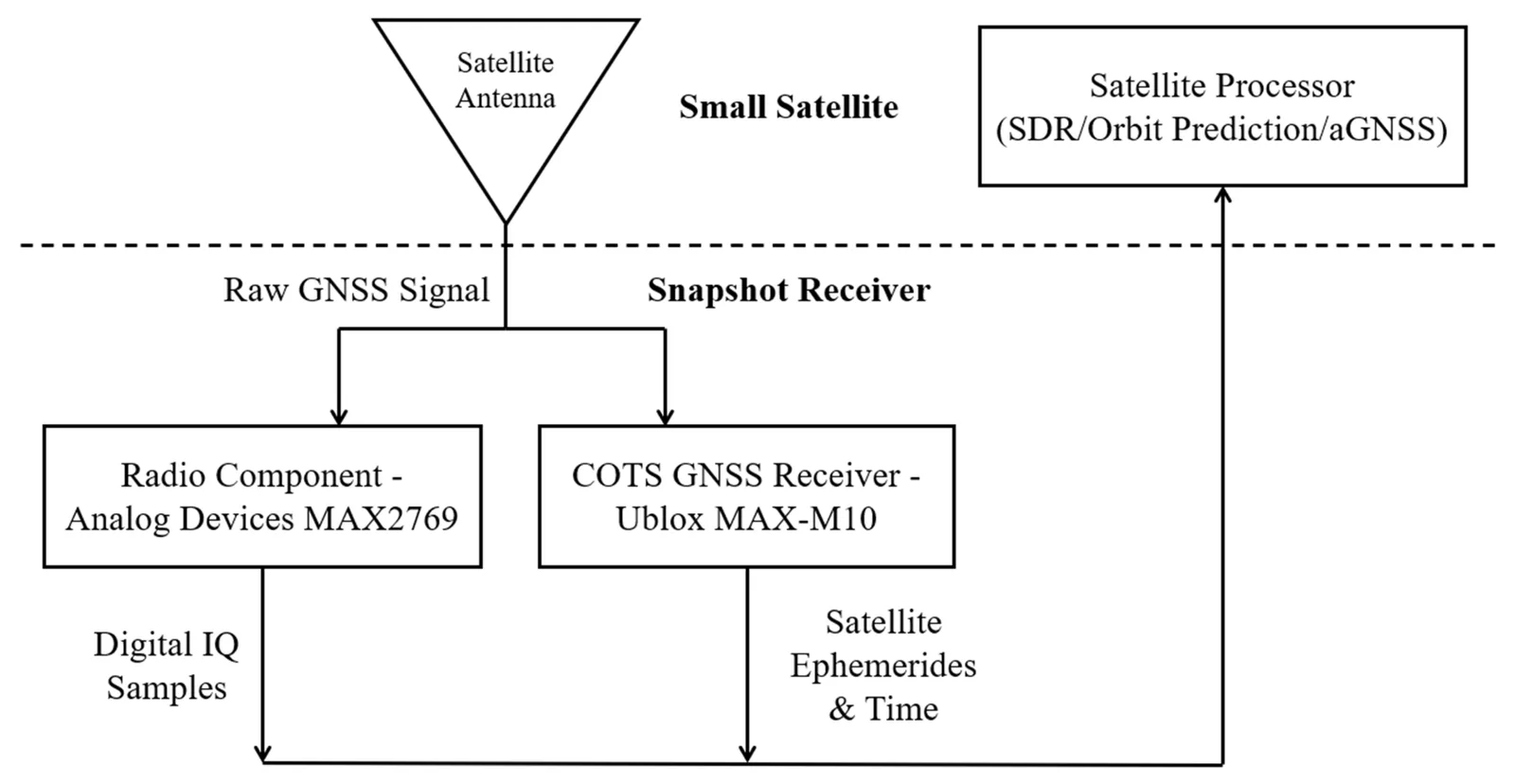
Block diagram of the snapshot receiver architecture without an independent processor. Measurements are passed to the main satellite processor for computation of aGNSS solutions.

With access to digital IQ samples, satellite ephemerides, a time solution, and the location from the orbit prediction methods, aGNSS solutions can be computed. This process, along with the initial collection and computation of a traditional GNSS solution to initialize the system, can be done either by a separate processor specifically for the snapshot receiver or through a processor already included in the overall satellite system. This choice is entirely dependent on the needs and capabilities of the existing satellite system, and the snapshot receiver presented in this paper is designed to be agnostic to this choice. As such, two system diagrams are presented here, one that includes a separate processor that passes along the resulting Position, Navigation, and Timing (PNT) solutions to the main satellite processor, and one within which the IQ samples, ephemerides, and timing information are passed directly to the main satellite processor, which does all the computations for the system. Block diagrams for both systems are presented respectively in Figure 1 and Figure 2 below.

## 3. Orbit Determination and Prediction Methodologies

### 3.1. Initial Orbit Determination

To obtain an aGNSS solution using GPS, a position estimate within 100 km and a time estimate within 1 min are required [10]. While a traditional snapshot receiver is unable to determine a position or time alone, a longer collection of GNSS signals can be used for this function. To determine the satellite’s orbit, an initial collection of 37 s of GNSS signal raw IF data is collected at a 2 Msps sampling rate with an Analog Devices MAX2769, a low-power radio component. This length of collection allows for a traditional GPS solution from L1 C/A signals, providing position, velocity, and timing solutions. This collection is pushed through a Software-Defined Radio (SDR) code created by the RF and SatNav Lab at the University of Colorado Boulder, generating accurate positions and velocities at a 1 Hz rate throughout the collection. In addition, the GNSS timing information is decoded and saved, allowing for accurate estimates of time at future epochs for use in the snapshot receiver. Using the results of position and velocity, orbital elements can be generated using traditional methods described in [13]. First, since these values are initially represented in the Earth-Centered Earth-Fixed (ECEF) frame, they must be transformed into an inertial frame such as the Earth-Centered Inertial (ECI) frame to calculate orbital elements. Following conversion and conventional methods to determine orbital elements, the initial orbit of the satellite is determined with a certain margin of error. To correct the errors in these measurements, and to better determine the orbit, a least-squares solution using finite differencing is implemented. The theoretical background for this solution is based on the work in [5,14]. The measurements of position and velocity comprise the six-element state-space being corrected in the least-squares solution. For a single collection, this least-squares solution allows for the incorporation of all measurements to be utilized and corrected; multiple collections can similarly be used to correct for the long-term effects of the LEO environment.

### 3.2. SGP4 Orbital Prediction

To determine the satellite’s position at future epochs, an orbital prediction method must be implemented. For this, we have selected the SGP4 propagator based on its widespread use and documentation. SGP4 incorporates various perturbations, including those based on gravitational effects from Earth’s shape and the force of drag [15]. In addition, when an orbit’s period is greater than 225 min, SGP4 incorporates lunar and solar gravitational effects [15]. In total, SGP4 provides a well-established propagation system that incorporates many of the perturbations experienced in the LEO environment. As the SGP4 propagator has gone through various updates throughout its history, we base our implementation on the methods and code presented in the paper “Revisiting Spacetrack Report #3: Rev 2” by Vallado et al. [5]. Much of the code utilized for SGP4 is based on the work of [16], which adapted the work in [5] to a MATLAB (version R2025a) implementation. To properly utilize the SGP4 propagator, corrections must be made to the calculated orbital elements, including the conversion of osculating elements to mean elements. For this, the first-order mapping based on Brouwer-Lyddane, as presented in [17], is utilized to calculate the mean elements suitable for use in the SGP4 propagator. As the SGP4 propagator is designed to be used with TLEs as its input, we utilize the corrected orbital elements and additional parameters to generate a pseudo-TLE for use in SGP4 propagation. Among these additional parameters are the drag-like Bstar term and the first and second derivatives of mean motion. These terms are dependent on the geometry, weight, and orbit of the full satellite system and can be computed based on equations presented in [5]. These parameters will need to be determined and updated based on the full mission implementation.

### 3.3. Updating GPS Ephemerides

Another key factor in the development of an aGNSS solution is access to an updated GNSS ephemeris. While this information could be uplinked to the satellite from a variety of external sources, the ability for the system to determine the satellite ephemerides itself is desired. One method for the system to determine ephemeris information is to process SDR collections, as they are utilized to generate pseudo-TLEs for orbit prediction described above, wherein the ephemerides are directly decoded from the satellite signals. While this is effective, the collection and processing of this information requires processing time and power. As explained in Section 2.1, a LEO satellite only has visibility of a GPS satellite for around 15 min, depending on the orbit. To acquire ephemerides for all satellites of the GPS constellation, multiple collections would need to be taken at this interval for an entire orbit. As GPS ephemerides update every 2 h, these collections would have to be repeated often to keep ephemeris updated, wasting power and time on computation. Instead of power-hungry SDR collections, a traditional Commercial Off-The-Shelf (COTS) GNSS receiver can be leveraged to decode the ephemeris. Many receivers can output ephemeris information for the satellites they acquire, but a low-power option is desired. While traditional COTS receivers are designed to not output position solutions in a LEO environment due to altitude- and velocity- dependent operational limits, the ephemeris information can be decoded and output by a variety of COTS receivers. These ephemeris collections must still be performed multiple times throughout the orbit to collect all GPS satellites, but this method uses much less power and computation time than standard decoding. Crucially, using a COTS receiver allows for the ephemeris collection to occur in real time, allowing for aGNSS solutions to use satellites that recently came into view throughout the orbit. In addition, this COTS receiver allows for time updates synchronized to the GPS system during ephemeris collection. This allows for the system time to be updated for implementation in the aGNSS solution. These benefits make the inclusion of a COTS receiver extremely valuable in enabling aGNSS solutions for the lowest amount of power consumption possible.

## 4. Analysis of Computation and Power Consumption

One major constraint for calculating computationally intensive GNSS solutions on a small satellite is the availability of power. Finding an appropriate hardware configuration that is both power-efficient and has sufficient computational ability is crucial for enabling positioning. For testing total computing time and power draw, three candidates from the Raspberry Pi family were selected: the Raspberry Pi 5, Raspberry Pi Zero 2 W, and Raspberry Pi Zero W. These devices were selected to cover a range of computational ability and power consumption while allowing for relatively simple deployment and testing of the SDR, SGP4, and aGNSS codebases when compared to other microcontroller platforms. Technical specifications for these devices are listed in Table 1. The Raspberry Pi family offers highly accessible and cost-effective solutions for low-cost CubeSat development, with spaceflight heritage demonstrated by missions such as GASPACS from Utah State University [18] and CubeSX-HSE from the Moscow Institute of Electronics and Mathematics at the National Research University Higher School of Economics [19]. It should be noted, however, that the Raspberry Pi family was utilized in this study as a representative hardware platform to validate the technical feasibility of the proposed conceptual research. The software-defined nature of this approach ensures that it can be readily ported to more power-efficient microprocessors, Field-Programmable Gate Arrays (FPGAs), or Application-Specific Integrated Circuits (ASICs), tailored to the specific mission objectives and stringent power budgets of various satellite architectures. Furthermore, as detailed in the architectural discussion in Section 2.2, the system provides the flexibility to utilize the existing satellite onboard computer for signal processing, thereby eliminating the need for a dedicated processor within the receiver module and further optimizing the system’s SWaP-C profile.

As a note, the codebases used for these experiments are entirely based on Python 3, which is beneficial for ease of deployment and testing, but are not focused on time efficiency. These results serve as a solid baseline, but there is still much room for improvement. Various avenues such as switching from the interpreted Python language to a compiled language such as C or C++, implementing the use of Single Instruction, Multiple Data (SIMD) instructions, or designing a custom ASIC or FPGA are likely to provide notable performance gains, with a potentially lower energy footprint or peak amperage. In many cases, however, these improvements would require complete ground-up rewrites of the codebases used for these experiments and are likely only reasonable when a real-world deployment of a similar system is required.

### 4.1. Methods

To find the power and performance characteristics, each device was powered by a variable DC power supply with a multimeter connected. Amperage and voltage were recorded, as well as the computation time, using methods implemented in software. These characteristics were recorded separately for the three major components of the positioning system: the full SDR code, SGP4 propagation, and an aGNSS implementation.

For the RF and SatNav Lab‘s Python SDR codebase, tests were conducted, with 37 s data files collected at 2 Msps. For the SGP4 Orbital Prediction, a test dataset taken from a COTS receiver was used to propagate positioning once every second for a full day using a Python codebase [20] by David Vallado. Similarly, a longer sample of the same dataset used to test the SGP4 propagation was used to test the Python aGNSS implementation for 144 solutions. This number was selected, as discussed later in Section 5.4, to provide an optimal fit span for SGP4 orbital determination. Results from these tests are shown in Table 2, Table 3 and Table 4.

**Table 1:** Raspberry Pi technical specifications [21,22,23].

Device	Processor	Memory
Raspberry Pi 5	ARM Cortex-A76 4 cores at 2.4 GHz	2 GB LPDDR4X SDRAM
Raspberry Pi Zero 2 W	ARM Cortex-A53 4 cores at 1 GHz	512 MB LPDDR2 SDRAM
Raspberry Pi Zero W	ARM 1176JZF-S 1 core at 1 GHz	512 MB LPDDR2 SDRAM

**Table 2:** Duration and amperage for 37 s GNSS solution.

Device	Compute Duration (mm:ss)	Peak Amperage	Total Energy Consumption (Wh)
Raspberry Pi 5	01:11	0.95 A	0.085 Wh
Raspberry Pi Zero 2 W	13:28	0.33 A	0.331 Wh
Raspberry Pi Zero W	78:48	0.20 A	1.293 Wh

**Table 3:** Duration and amperage for 1 day of SGP4 propagation at 1 Hz update rate.

Device	Compute Duration (mm:ss)	Peak Amperage	Average Time per Propagation	Total Energy Consumption (Wh)
Raspberry Pi 5	00:11	0.77 A	0.152 ms	0.012 Wh
Raspberry Pi Zero 2 W	01:43	0.25 A	1.318 ms	0.033 Wh
Raspberry Pi Zero W	10:42	0.14 A	7.112 ms	0.107 Wh

**Table 4:** Duration and amperage for 144 aGNSS computations.

Device	Compute Duration (mm:ss)	Peak Amperage	Average Time per Solution	Total Energy Consumption (Wh)
Raspberry Pi 5	00:03	0.78 A	22.769 ms	0.003 Wh
Raspberry Pi Zero 2 W	00:30	0.25 A	205.458 ms	0.010 Wh
Raspberry Pi Zero W	03:03	0.19 A	1271.142 ms	0.042 Wh

Across the board, the results indicate that the faster devices are more power efficient overall but require higher peak current. The Raspberry Pi 5 yields the quickest computing times for every task, at the cost of having the highest peak current requirement of 0.95 A. On the other hand, the Raspberry Pi Zero W consistently operates with the lowest peak current, while taking the longest and being the least power efficient. Choosing the ‘best’ microcontroller is not possible without knowing all the details of the host satellite and mission details. The most optimal configuration varies from case to case.

### 4.2. Total System Computation Energy Assessments

With these measurements, we can estimate the energy consumption and computation profiles of the suggested PNT implementations discussed for LEO satellites, as well as any other configuration using these major elements. The examples shown here serve as a demonstration and are meant to be a guideline rather than a final implementation.

**Figure 3:**
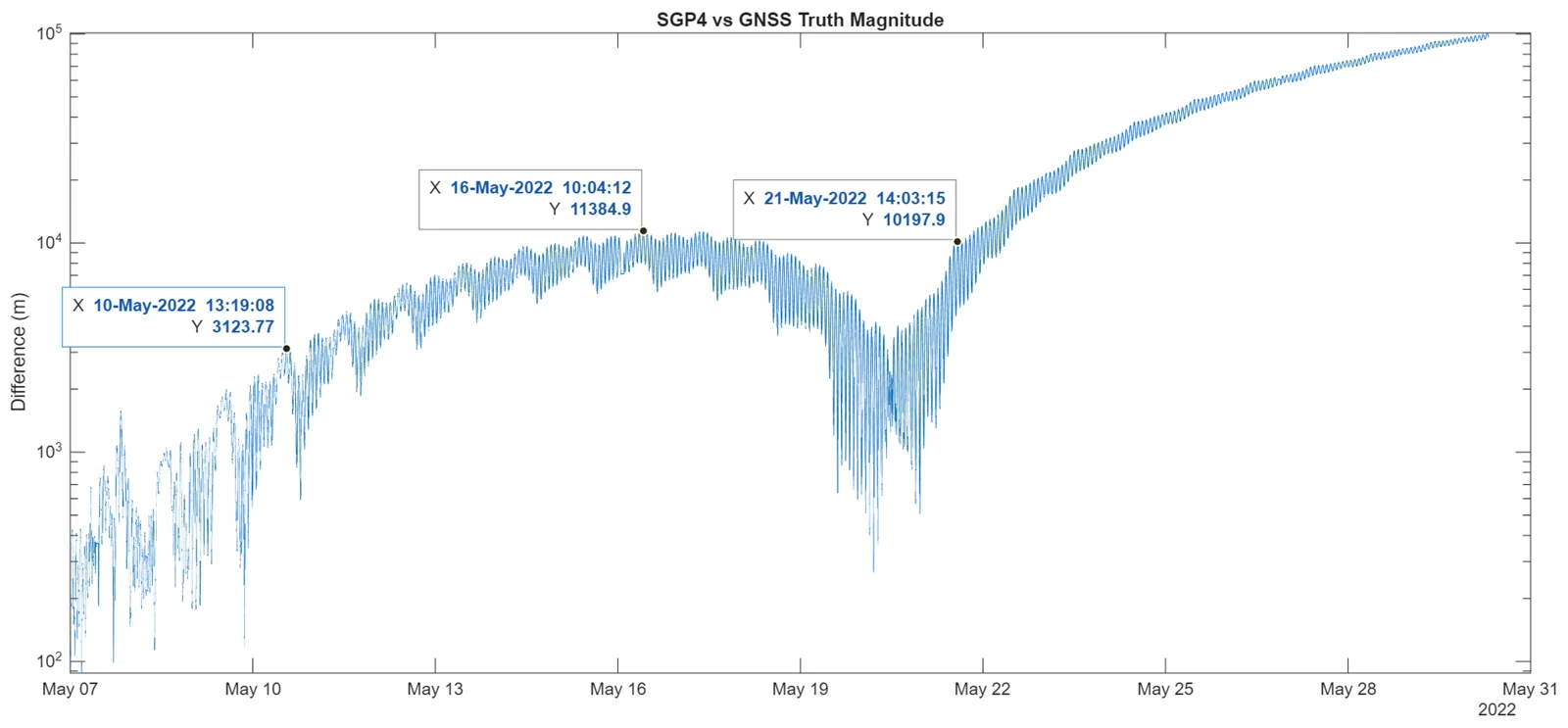
Plot of the SGP4 accuracy over the period of 3 weeks using a fit span of three 40 s collections, each separated by 3 h. Key points are given in meters.

The implementation that will be discussed in Section 5.1 suggests using three collections of 40 s of raw IF data from a LEO satellite, followed by SGP4 propagation, until the desired accuracy is no longer met. After 2 weeks of propagation, accuracy decreases at a more drastic rate, shown in Figure 3, so that will be the cycle time for this example. Using this, total power statistics can be computed by summing the power draw for three SDR computations and 14 days of SGP4 propagation, shown in Table 5.

The implementation in Section 5.3 instead suggests using one initial collection of 40 s of raw IF data from CYGNSS, followed by 144 aGNSS solutions, finishing with 2 weeks of SGP4 propagation before accuracy degrades much like the previous method. The resulting power draw for each device is shown in Table 6.

Based on these measurements, the aGNSS implementation reduces computational-processing energy consumption by approximately 40% compared with the non-aGNSS implementation across all tested devices. This energy reduction is achieved by reducing the number of times the full SDR processing is executed, in favor of the more lightweight aGNSS code. These results validate the practical advantages of the aGNSS solution, demonstrating improvements in both positioning accuracy and energy efficiency compared to the non-aGNSS method. It should be noted that the approximately 40% reduction applies only to computational-processing energy. In a practical receiver, additional energy consumption associated with the RF front end, the COTS GNSS receiver, processor idle/sleep/wake-up operation, data storage and transfer, clocking, and other supporting electronics would reduce the relative improvement at the overall receiver or system level. Therefore, the reported approximately 40% reduction should be interpreted as a computational-processing energy estimate rather than a 40% reduction in total receiver energy consumption.

## 5. Fit Span and Accuracy Discussion

### 5.1. Fit Spans Using 40 s Collections

With the ability to predict satellite states in future epochs, the opportunity to perform aGNSS solutions is realized. However, many questions remain as to the full system implementation. For instance, the accuracy of the propagation methods must be investigated to determine how long the generated pseudo-TLE can be used. As aGNSS using GPS requires a position estimate within 100 km, the propagation must be within this range to be viable. However, based on testing with a GNSS simulator, aGNSS position solutions in LEO with our implementation become inconsistent past an 80 km error due to the dynamics in the LEO environment; so, this limit will be imposed to ensure reliability of the system. If a position estimate is outside of this range, a position solution is not computed, or an incorrect solution may be computed. It is important to note that the position estimate from this propagation could then be used as the initial estimate for aGNSS, which in turn enables a refined final position estimate. For reference, the prior study in [3] estimated a 3D RMS positioning accuracy of approximately 19 m for a spaceborne snapshot GNSS architecture. As such, while the accuracy of the propagation scheme itself can reach errors of up to 80 km, if this is within this bound, an aGNSS solution could then be implemented and result in a much higher-accuracy solution.

Determining the accuracy of propagations is key to understanding the availability of aGNSS solutions. To verify the accuracy of the propagations, GPS data collected from a Cyclone Global Navigation Satellite System (CYGNSS) satellite is used. CYGNSS is a constellation comprised of eight satellites in LEO designed to utilize GNSS for remote sensing applications. For our purposes, continuous GPS position, velocity, and timing (PVT) data from a CYGNSS satellite for a period of a month was collected to describe the motion of a real LEO satellite. We utilized 40 s subsets of the PVT data to estimate the initial orbit state and to propagate forward, comparing it to the truth results of the full GPS collection.

**Figure 4:**
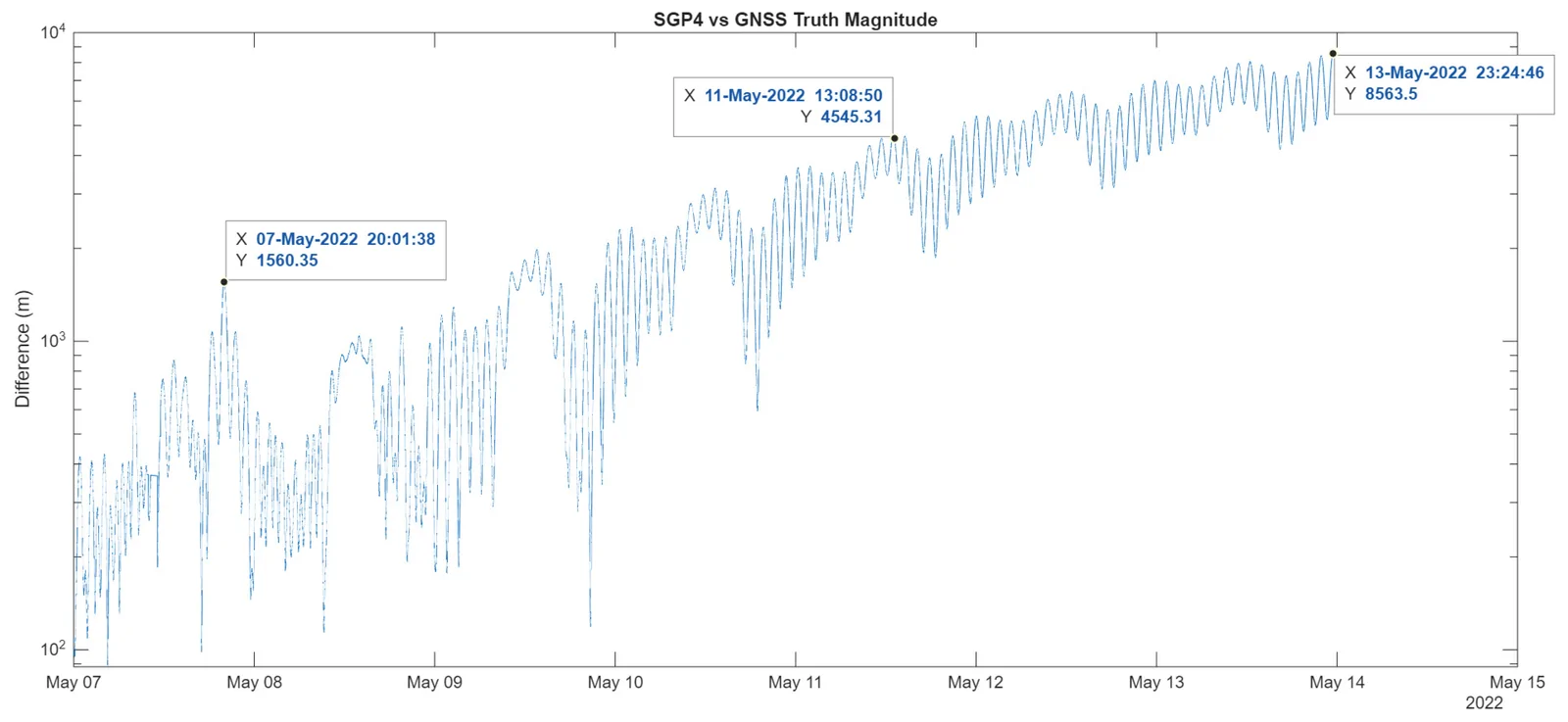
Plot of the SGP4 accuracy over the period of a week using a fit span of three 40 s collections, each separated by 3 h. Key points are highlighted to illustrate the time elapsed and the resulting accuracy of SGP4 propagation in meters.

The period in which position estimates are in the 80 km range is dependent on the fit span and the update rate of the measurements used to generate the pseudo-TLE, as well as the methods to combine these measurements. A key limitation with the full GNSS collections used for orbit determination is the power and time required to process the PVT solutions. With these constraints, three collections of 40 s of GNSS data from CYGNSS spread out by 3 h between collections are used to generate a TLE for propagation. Based on the analysis of computation time in Section 4, this fit span is representative of the capabilities of the full system architecture. To integrate these measurements, a least-squares application is utilized based on the methods presented in [14]. The resulting fitted parameters from the least-squares implementation are propagated at a 1 Hz rate for three weeks through SGP4, beginning on 7 May 2022. A graph of the error between the SGP4 predictions compared to the truth GNSS position for periods of one week and three weeks are provided in Figure 3 and Figure 4 respectively, utilizing a logarithmic scale.

As depicted in the figures above, the accuracy of SGP4 propagation with this fit span remains under 12 km for two weeks of propagation. In addition, the accuracy remains under 5 km for four days and remains near 1 km accuracy for nearly a day. While providing these accuracies alone may be sufficient for some applications, they also allow for aGNSS solutions to be computed for this entire period. Overall, a small collection of measurements enables accurate propagations for 2 weeks, potentially longer if the dynamics of the satellite remain constant throughout the propagation period.

### 5.2. The Importance of the Drag Parameter

A key aspect of note is the requirement for an accurate estimate of the SGP4 drag parameter, $B^{*}$. For the tests above, $B^{*}$ was manually selected to achieve the best propagation results, without knowledge of its true value. In an operational implementation, however, $B^{*}$ could be estimated jointly with the orbital elements using available position and orientation measurements. Several studies have investigated the estimation of the SGP4 drag parameter. An SGP4 differential-correction approach has been presented in which $B^{*}$ can be estimated together with the orbital elements through batch least squares [14]. A finite-difference-based extended Kalman filter has also been proposed for real-time estimation of the TLE parameters, including $B^{*}$, from positioning data [24]. The present study does not implement such an estimator and instead assumes that a sufficiently accurate $B^{*}$ estimate is available. Under this assumption, this section analyzes the sensitivity of the propagation performance to errors in $B^{*}$ and emphasizes the importance of accurate drag parameter estimation.

**Figure 5:**
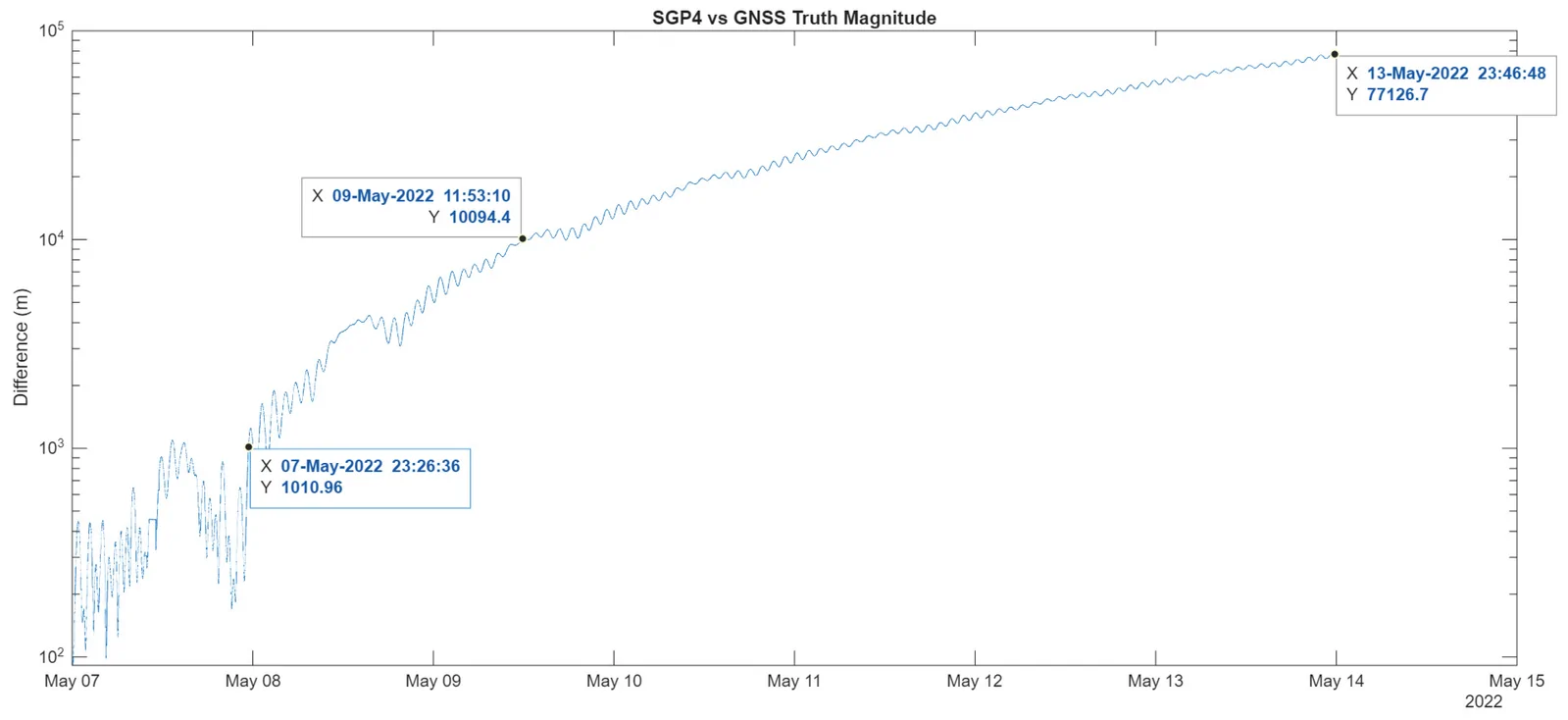
Plot of the SGP4 accuracy over a 1-week period with 2× the drag parameter, using a fit span of three 40 s collections separated by 3 h each. Key points are given in meters.

**Figure 6:**
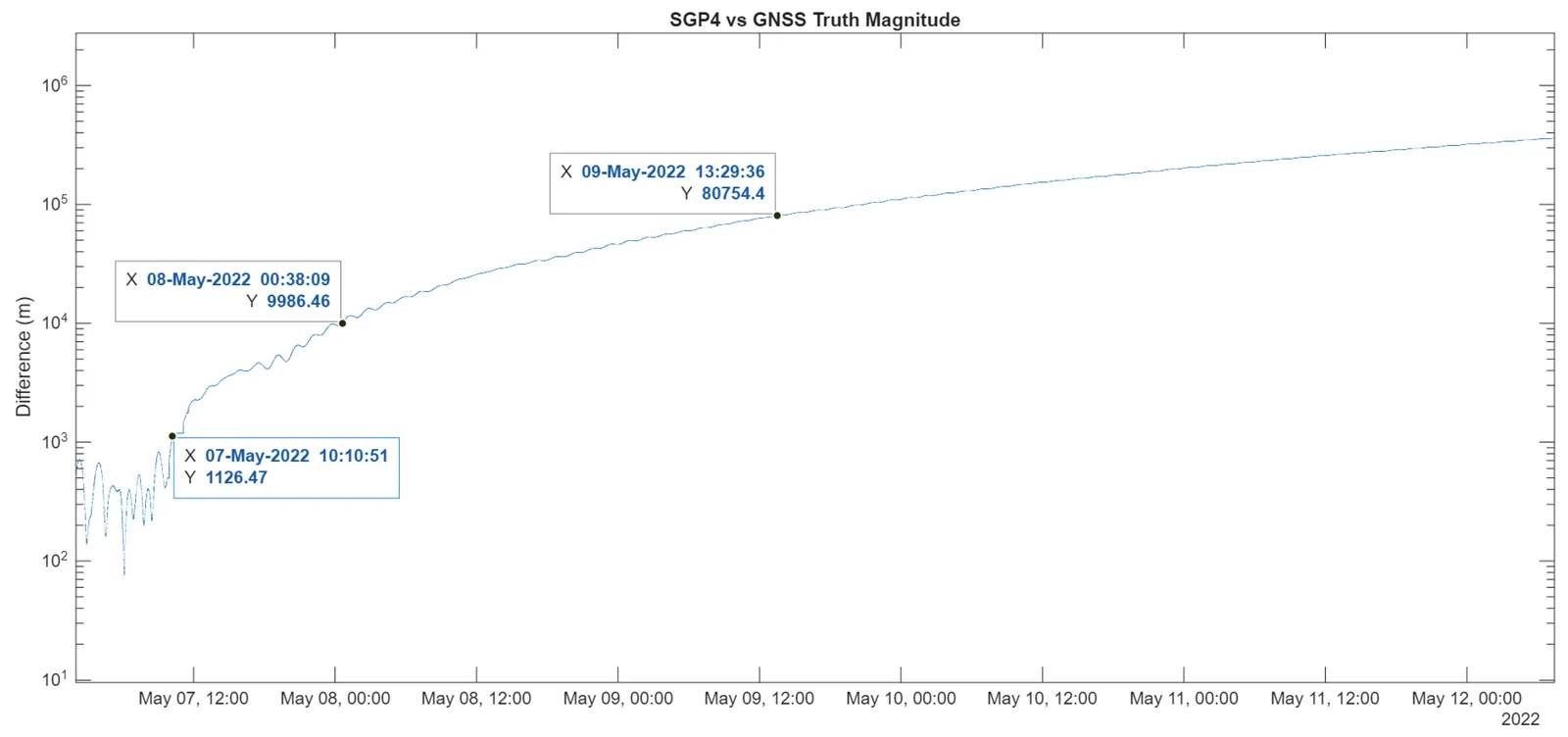
Plot of the SGP4 accuracy over a 1-week period with 10× the drag parameter, using a fit span of three 40 s collections separated by 3 h each. Key points are given in meters.

To better characterize the importance of the drag parameter, tests are conducted with the same data above, but by multiplying the drag parameter by 2 and 10 times its determined value. Figure 5 and Figure 6 below show the week-long propagation with a drag parameter of 2 times and 10 times the value determined in the tests above, respectively.

As illustrated in the figures above, an inaccurate drag parameter can significantly degrade the accuracy of SGP4 propagation. With a drag parameter 2× the estimated value, the estimate remains within 10 km accuracy for only 2.5 days, significantly shorter than the near 2-week period with a correct drag. When the drag parameter is 10× its estimated value, the accuracy degrades faster, staying within 10 km for only a day. While the accuracy remains within 80 km for around a week in the 2× case and for 2.5 days in the 10× case, enabling aGNSS solutions for these periods, this accuracy is significantly worse than with a proper drag parameter. While the mechanics of the estimation of drag parameters are not discussed in this paper, it is key to note its importance on both the short-term and long-term accuracy of SGP4 propagation. For the most effective incorporation of the system of this paper, a reasonably accurate drag parameter is needed, allowing for the highest base accuracy and most consistent ability to utilize aGNSS solutions if desired.

### 5.3. Leveraging the Availability of aGNSS Solutions

**Figure 7:**
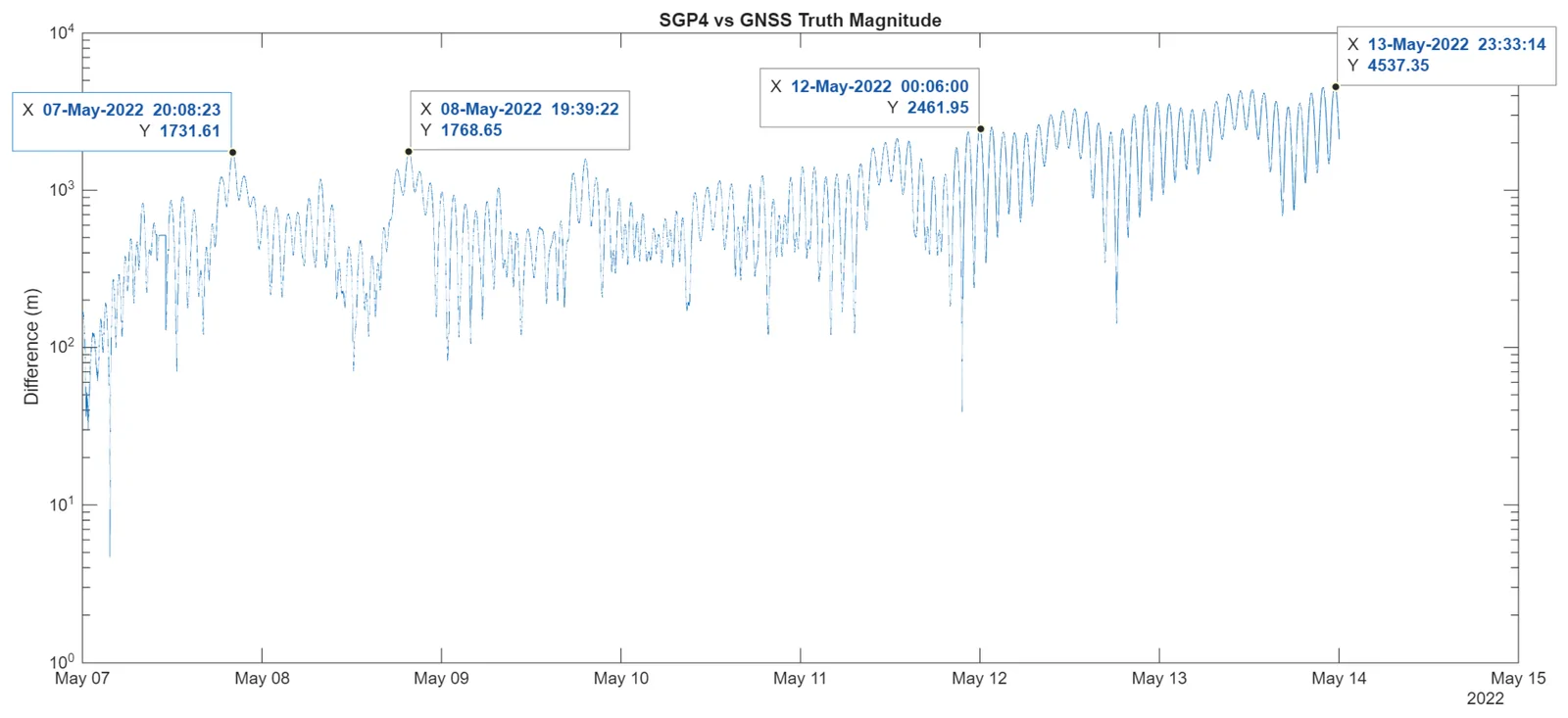
Plot of the SGP4 accuracy over the period of a week using a fit span of 144 aGNSS measurements over a 2-orbit period. Key points are given in meters.

**Figure 8:**
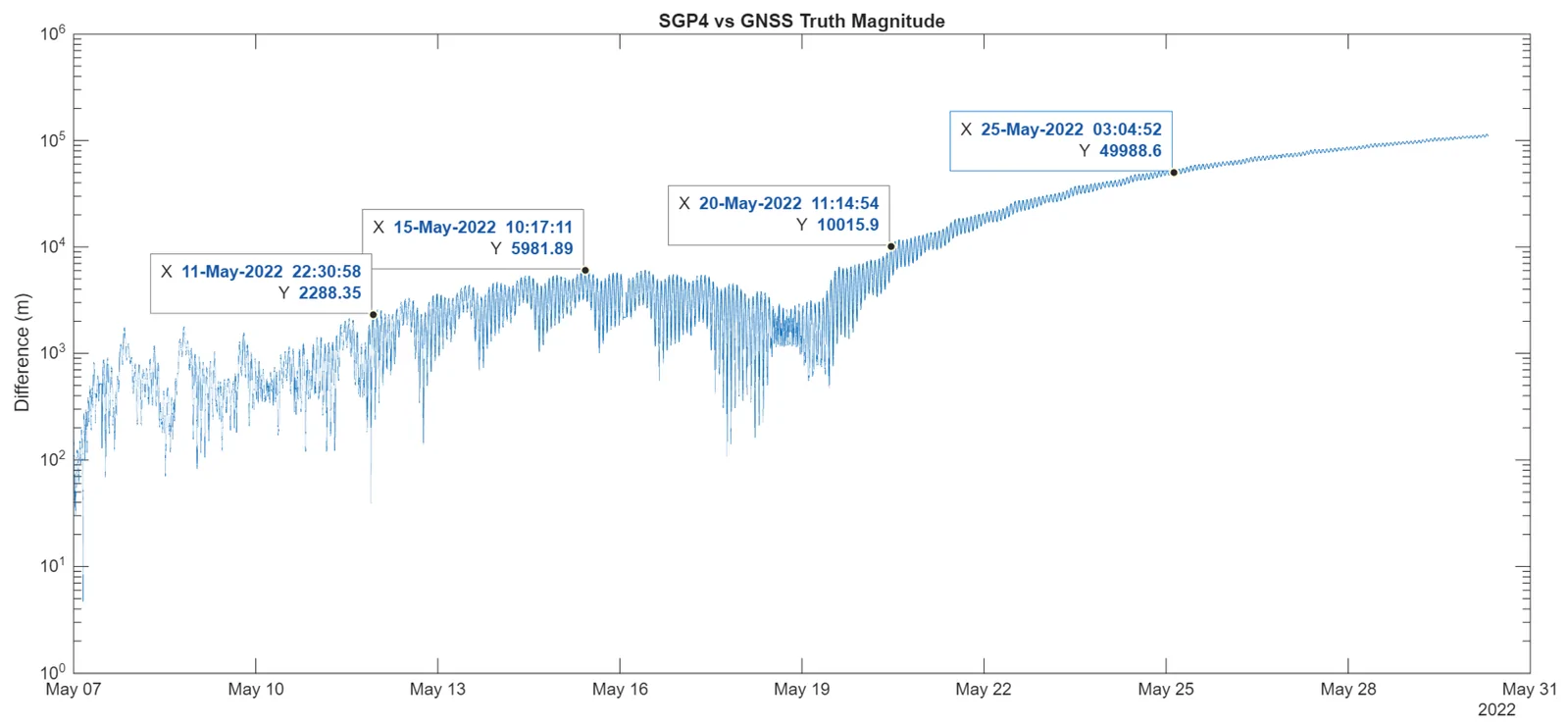
Plot of the SGP4 accuracy over the period of 3 weeks using a fit span of 144 aGNSS measurements over a 2-orbit period. Key points are given in meters.

While these full collections allow for aGNSS solutions for an extended period, by leveraging the assisted solutions, repeated lengthy collections of IF data can be avoided and higher accuracy can be achieved. According to research done in [14], a quality fit span for SGP4 orbit determination is described to be 72 collections per orbit over a two-orbit period, resulting in the total span of 144 collections used below. By using one initial IF collection, a propagation sufficient to perform aGNSS over this span is obtained. With the customizable variability of aGNSS solutions, measurements are easily collected to provide this fit span. To integrate these measurements, a least-squares application is utilized, as described in [14]. By integrating aGNSS solutions within the fit span, multiple costly IF collections are avoided, increasing the power efficiency of the system. Furthermore, the resulting accuracy of orbit determination is increased. To test this, the same data from a CYGNSS satellite above is used, but with this improved fit span simulating the availability of aGNSS measurements. The resulting accuracy is illustrated in Figure 7 and Figure 8 below, with propagation times of 1 week and 3 weeks, respectively.

With this improved fit span utilizing aGNSS solutions, the accuracy of propagation is improved compared to the original implementation above. The accuracy with this fit span remains under 10 km for roughly 2 weeks, while remaining under 6 km for much of this period. In addition, the accuracy remains under 2.5 km for 5 days, a major improvement in the short-term propagation from the prior implementation, which had a 5 km error by this point. Much like the previous implementation, accuracy rapidly degrades after around 2 weeks. However, for at least 2 weeks, the accuracy of an aGNSS fit span is greatly improved compared to the full IF collections, particularly in the short-term propagation.

**Figure 9:**
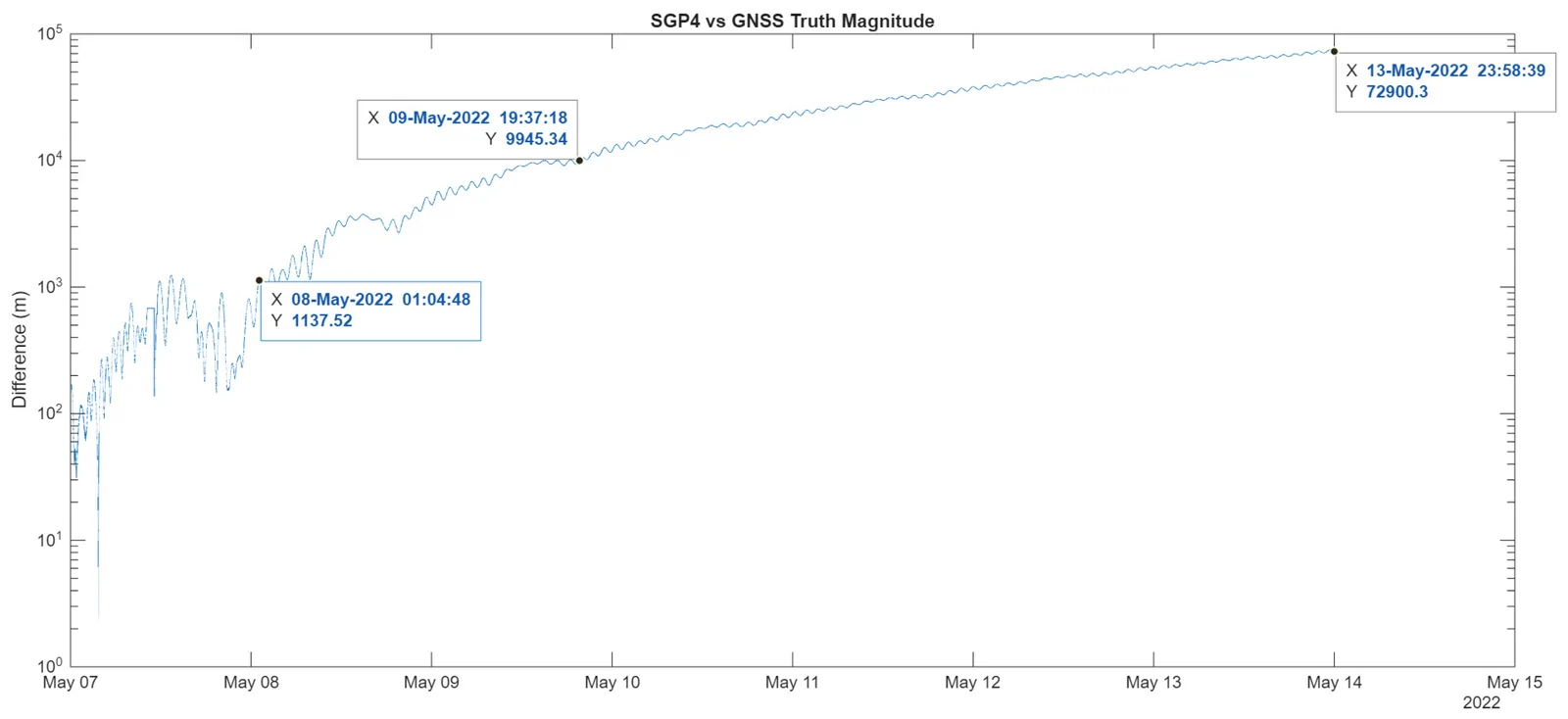
Plot of the SGP4 accuracy over a 1-week period with 2× the drag parameter, using a fit span of 144 aGNSS measurements over a 2-orbit period. Key points are given in meters.

**Figure 10:**
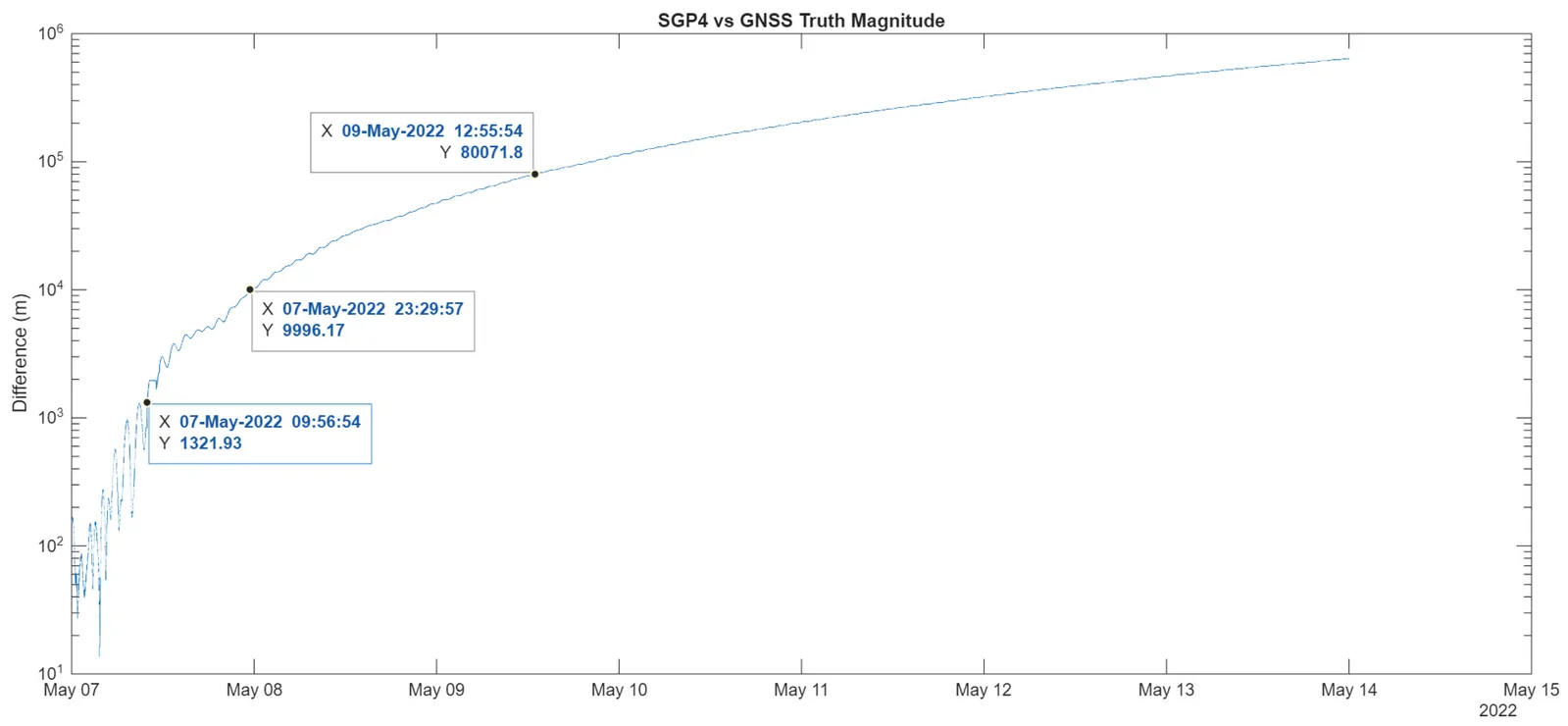
Plot of the SGP4 accuracy over a 1-week period with 10× the drag parameter, using a fit span of 144 aGNSS measurements over a 2-orbit period. Key points are given in meters.

As noted before, an accurate estimation of the drag parameter can have major implications for the accuracy of SGP4 propagation. The same drag parameter used in the original tests at the beginning of this section was used for the tests in Figure 7 and Figure 8, which were manually selected to achieve the best accuracy. A similar study is performed by again varying this drag parameter by 2× and 10× its value to illustrate the importance of accurate estimation. Figure 9 and Figure 10 below show the week-long propagation with a drag parameter of 2 times and 10 times the value determined in the tests above, respectively.

As displayed in the Figures above, a similar effect is seen with this improved aGNSS fit span compared to the original fit span. With 2× the drag parameter, the accuracy of propagation degrades significantly, with a 10 km error being reached in under 3 days compared to the near 2 weeks with a proper drag parameter. This further shows the importance of an accurate drag parameter for accurate SGP4 propagation. However, an 80 km accuracy is achieved for nearly one week with this inaccurate drag parameter, which would enable accurate aGNSS solutions throughout this period. When the drag parameter is off by an order of magnitude, the accuracy of propagation is significantly worse. With 10× the drag parameter, a 10 km error is reached in just under a day, and an 80 km error is reached in 2.5 days. This means that the SGP4 propagation is simply too inaccurate to trust alone, but aGNSS solutions are still possible for short periods.

Overall, it is clear that an accurate drag parameter is crucial to ensure accurate SGP4 propagation. With an incorrect drag parameter, the accuracy of propagation degrades significantly, quickly becoming less accurate than a traditional TLE approach. However, the ability to perform aGNSS solutions remains, even with a poor estimate of drag. This enables refined position estimates at customizable output rates for the duration of these periods. While a proper estimation of the drag parameter could result in a fit span being sufficient for aGNSS solutions for nearly three weeks, with aGNSS measurements available, new fit spans could be recursively computed throughout. These updates to fit spans would enable continuous access to aGNSS solutions throughout the lifetime of the satellite mission. In addition, even with poor estimates of drag, aGNSS solution availability is enough to update fit spans for continuous operation, notably, at a more frequent update rate.

### 5.4. aGNSS Fit Spans

Another key aspect of propagation accuracy is the choice of the propagation fit span. For this, there are two main factors that affect fit performance: the overall length of the fit span and the interval of measurements throughout. Since aGNSS measurements can output position solutions at configurable rates, these choices can be made to optimize accuracy or power usage tuned to the needs of any CubeSat. To show the effects of these choices, we first explore the total duration of the fit span, comparing it to the CYGNSS data of a real satellite in LEO. Notably, these propagations feature the same estimate of the drag parameter as the tests at the beginning of Section 5.

**Figure 11:**
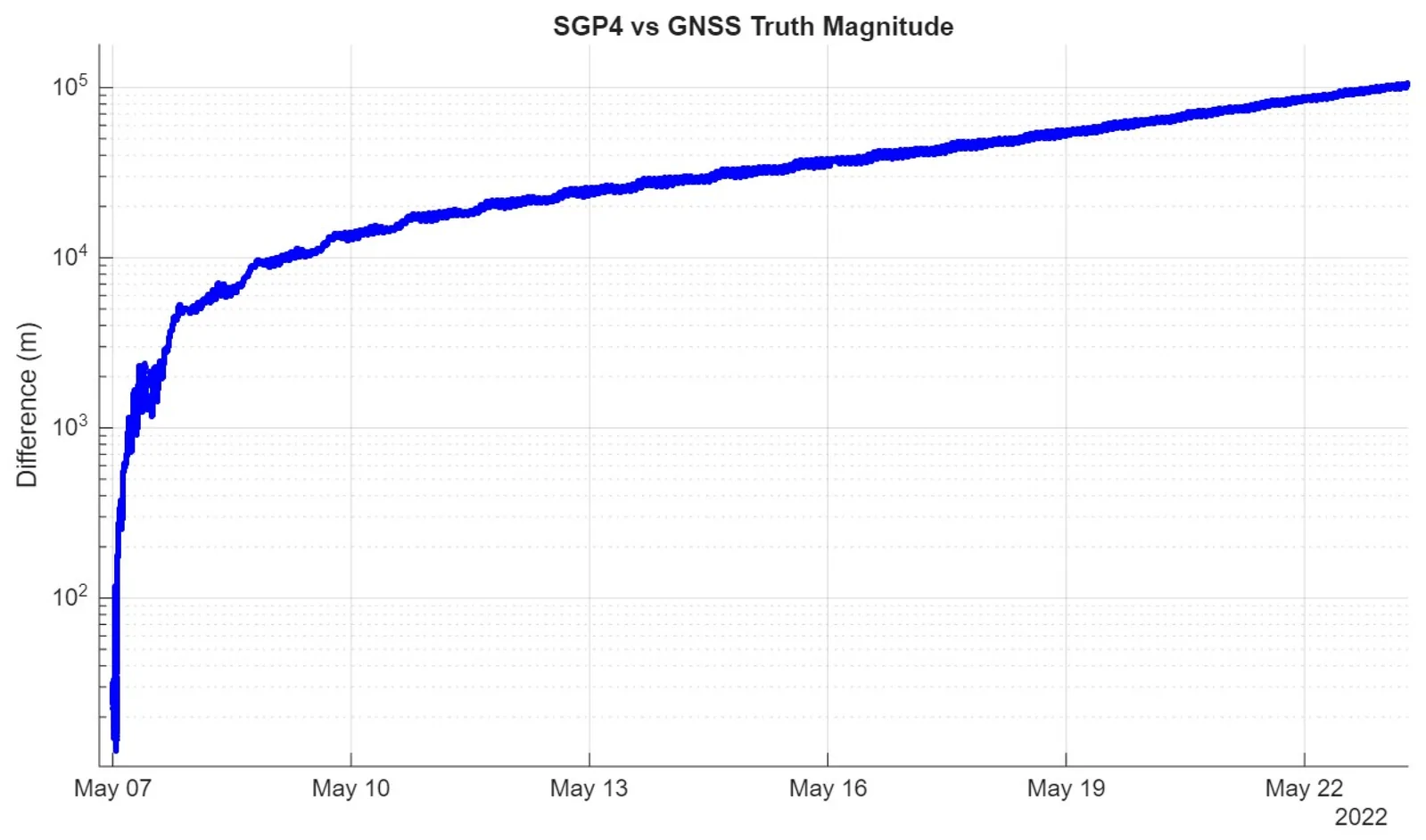
Plot of the SGP4 accuracy using a fit span of one hour.

To begin with, Figure 11 shows the accuracy of the SGP4 propagation using a fit span of one hour with measurements every 2 min. With less than an orbit period of data, the propagator diverges relatively quickly, reaching differences of over 10 km in under 3 days. However, the overall accuracy remains within the 80 km region required for aGNSS solutions for upwards of 2 weeks. This means that only a small amount of data is needed to allow for future aGNSS solutions for extended periods. This may allow for lower power usage, as the hardware only must run for a small number of measurements and propagation time, and the fitting is computationally easier. It is significantly less accurate than previously tested fit spans though, but the ability to receive aGNSS measurements at any defined interval is still realized for two weeks.

**Figure 12:**
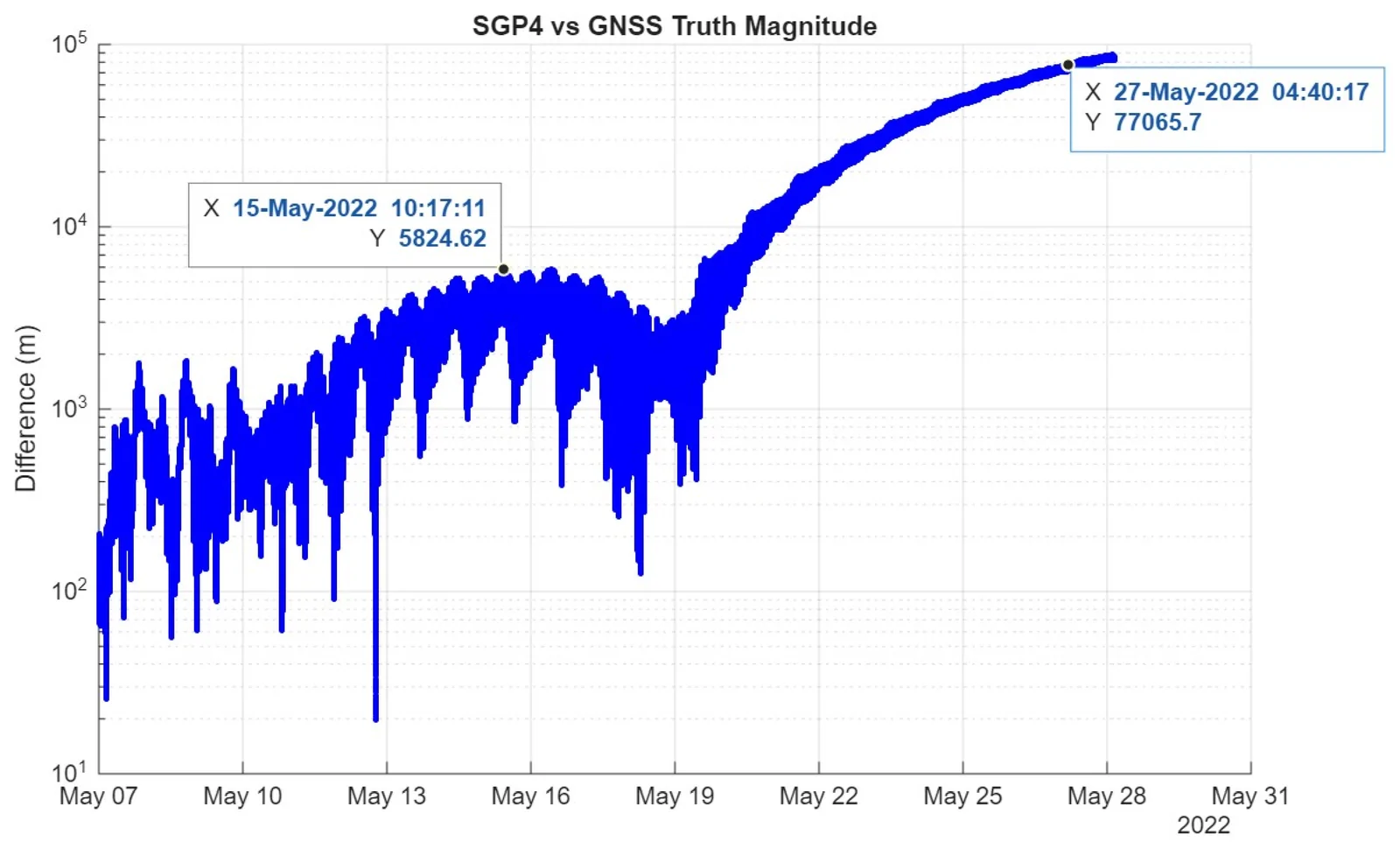
Plot of the SGP4 accuracy using a fit span of 6 h. Key points are given in meters.

To improve the short-term accuracy and long-term ability to perform aGNSS measurements, a longer fit span is desired. Earlier in Section 5, the fit span used a two-orbit period, which corresponds to roughly 3 h in a typical LEO orbit. This fit span was based on [14] and resulted in higher accuracy, particularly in the short term. To further this, Figure 12 shows the propagation accuracy using a longer fit span of 6 h. As shown in this figure, the propagation error remains within the 80 km window required for aGNSS measurements for up to three weeks, with performance similar to that obtained using the 3 h fit span. In addition, the short-term accuracy remains nearly unchanged. These results indicate that, for the cases considered in this study, a fit span corresponding to approximately 2–4 LEO orbits provides a sufficient observation arc to support long-term propagation for subsequent aGNSS solutions.

**Figure 13:**
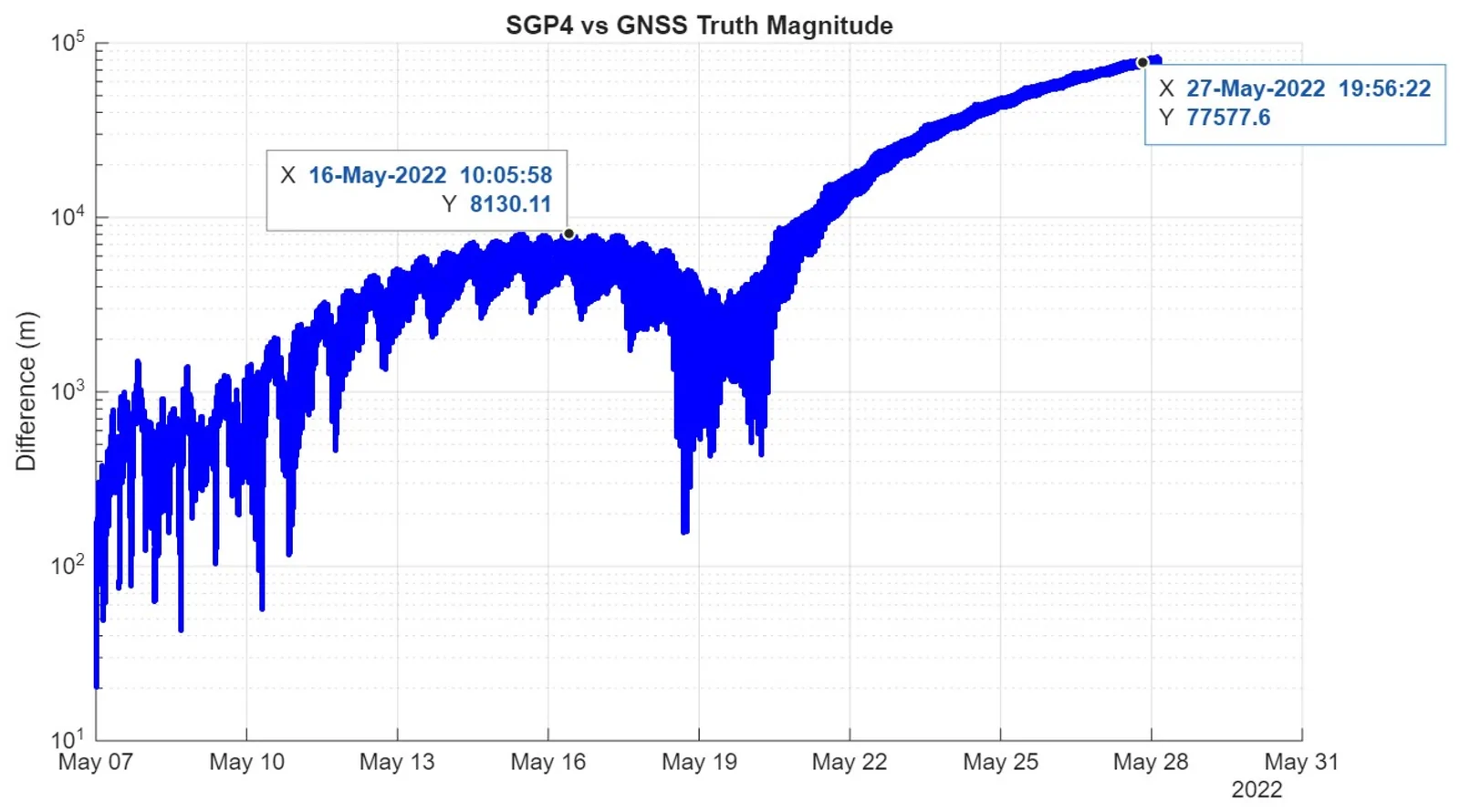
Plot of the SGP4 accuracy using a fit span of 12 h.

However, when the fit span is extended to 12 h, the propagation accuracy begins to degrade rapidly. This degradation can be attributed in part to the simplified nature of the SGP4 propagation model, for which model mismatch accumulates over longer propagation intervals. As the discrepancy between the propagated and measured states increases, the linearization used in the least-squares differential correction becomes less accurate, thereby degrading the performance of the batch solution. This effect can be seen in Figure 13 below, where a 12 h fit span results in errors larger than 80 km within 3 days. While this is significantly worse than the 3 weeks of other fit spans, it still shows that aGNSS solutions can be produced in the short term, which would allow for a new fit span to be calculated.

**Figure 14:**
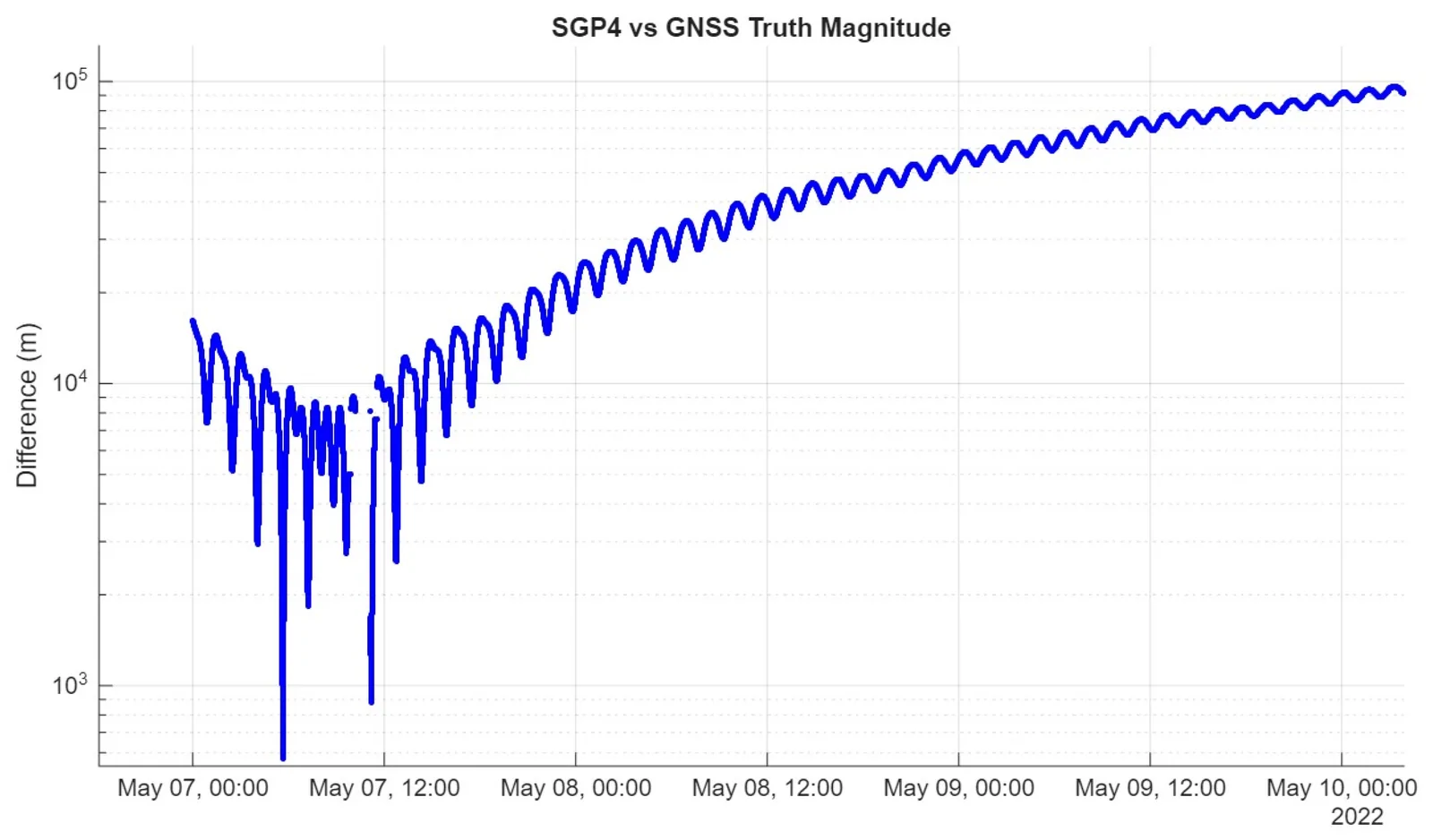
Plot of the SGP4 accuracy using a fit span of 6 h, with 1 h between each measurement. Key points are given in meters.

While the previous cases used measurements every 2 min, this also can be configured easily, resulting in potentially significant power savings. At the cost of some short-term accuracy, Figure 14 demonstrates a fit span of 6 h, with 1 h between each measurement. By using seven measurements total, a similar long-term accuracy is achieved compared to the fit with measurements every 2 min. This allows for an implementation in which the processor and receiver can be nominally inactive, briefly waking up every hour to collect small snapshots. This could enable a significant reduction in average power consumption while still providing an initial estimate suitable for subsequent aGNSS processing. These fits could then be repeated or implemented with Kalman filter approaches to enable continuously available aGNSS measurements at customizable rates and extremely low power.

### 5.5. Variability of Update Rates and System Implementation

**Figure 15:**
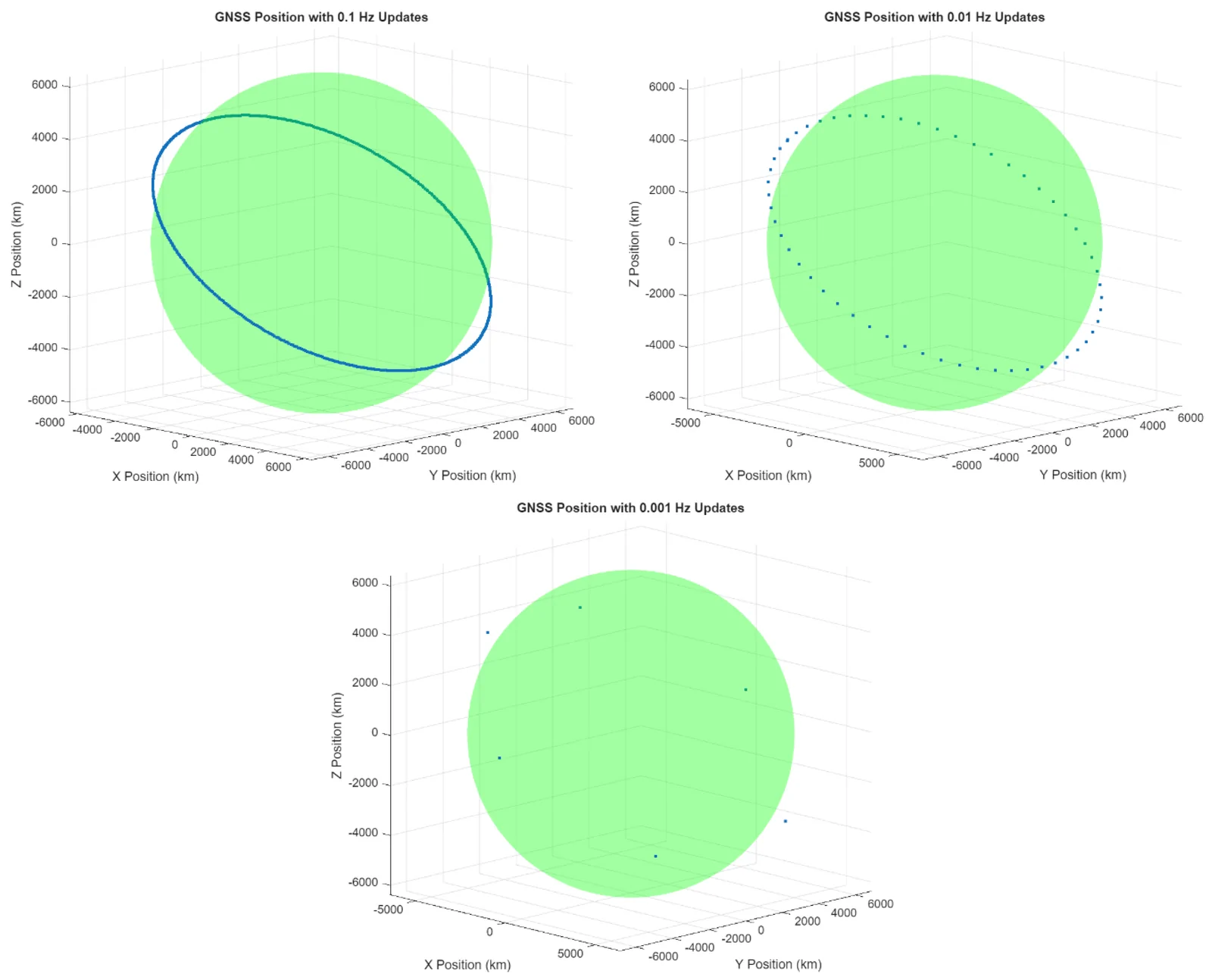
Plots of GNSS position solutions of a LEO satellite over one orbit with varying update rates. Update rates of 0.1 Hz, 0.01 Hz, and 0.001 Hz are included to display the levels of fidelity available with the system.

A major benefit of this system is the overall customization available in implementations to meet the needs of a variety of satellite systems. In using either aGNSS measurements or SGP4 propagation only, the output rates of position updates are entirely customizable to meet the needs of any mission. To help illustrate the effect of different update rates, visualizations are generated to show the fidelity generated over a single orbit of a LEO satellite. Update rates of 0.1 Hz, 0.01 Hz, and 0.001 Hz are chosen to display the variation of fidelity available with this system. It is difficult to effectively display update rates faster than 0.1 Hz; therefore, these are not included. With more measurements inherently comes more fidelity and the ability to more accurately determine the orbit of the satellite. Figure 15 below shows the results of these update rates over the course of one orbit.

## 6. Conclusions

In this paper, a novel design and methodologies for a low SWaP-C snapshot aGNSS positioning solution designed for CubeSats are presented. By strategically integrating a low-power COTS receiver for ephemeris acquisition with an SDR for short snapshot signal processing, the proposed system effectively addresses commercial altitude and velocity limitations while delivering high-fidelity PNT data. Orbit determination and propagation strategies are explored and implemented to enable aGNSS solutions for extended periods. The computational time and power draw for key system algorithms are tested on common representative processors. Accuracies of the propagation model are examined and validated using real GNSS data acquired from a CYGNSS satellite, demonstrating the ability to accurately predict the position of a LEO satellite. Errors of less than 10 km over a 2-week period are demonstrated with the ideal orbit-determination algorithm, providing sufficiently accurate initial estimates for aGNSS processing at configurable output rates. Impacts of the accurate estimation of the drag parameter with regards to propagation accuracy are explored, while the ability to perform aGNSS solutions remains for at least 2 days with poor drag estimation. While the long-term reliability of predictions is heavily contingent upon precise drag parameter estimation, the availability of frequent, high-accuracy aGNSS updates could enable orbital state estimates to be maintained recursively, even in dynamic conditions. Major functionalities are demonstrated, with needs for further exploration of a snapshot positioning module designed to meet the needs of a variety of LEO CubeSats or small satellites. Ultimately, these results suggest that the customizable framework has the potential to achieve substantially improved positioning accuracy over standard TLE-based tracking while supporting power-efficient implementation for CubeSats and other small-satellite platforms.

## Figures and Tables

**Table 5:** Comparison of power draw for system without aGNSS implementation.

Device	SDR Energy Consumption	SGP4 Energy Consumption	Total Energy Consumption (Wh)
Raspberry Pi 5	0.085 Wh/run · 3 runs	0.012 Wh/day · 14 days	0.420 Wh
Raspberry Pi Zero 2 W	0.331 Wh/run · 3 runs	0.033 Wh/day · 14 days	1.46 Wh
Raspberry Pi Zero W	1.29 Wh/run · 3 runs	0.107 Wh/day · 14 days	5.38 Wh

**Table 6:** Comparison of Power Draw for System with aGNSS Implementation.

Device	SDR Energy Consumption	aGNSS Energy Consumption	SGP4 Energy Consumption	Total Energy Consumption (Wh)
Raspberry Pi 5	0.085 Wh/run · 1 run	0.003 Wh	0.012 Wh/day · 14 days	0.252 Wh
Raspberry Pi Zero 2 W	0.331 Wh/run · 1 run	0.010 Wh	0.033 Wh/day · 14 days	0.811 Wh
Raspberry Pi Zero W	1.29 Wh/run · 1 run	0.042 Wh	0.107 Wh/day · 14 days	2.84 Wh

## Data Availability

The data that supports this paper is available from the corresponding authors upon reasonable request.
